# A novel pentavalent vaccine candidate completely protects against *Acinetobacter baumannii* in a mouse model of peritonitis

**DOI:** 10.1007/s00253-022-12231-3

**Published:** 2022-11-19

**Authors:** Yomna A. Hagag, Heba Shehta Said, Hany I. Kenawy, Ramadan Hassan

**Affiliations:** Department of Microbiology and Immunology, Faculty of Pharmacy, Mansoura University, Mansoura, 35516 Egypt

**Keywords:** *Acinetobacter baumannii*, Vaccine, Recombinant proteins, Capsular polysaccharides

## Abstract

**Abstract:**

*Acinetobacter baumannii* is considered as one of the most virulent and infectious organisms that have an increased ability to both evade host immune response and resist various classes of antibiotics, leading to life-threatening infections. Multiple virulence factors have been implicated in the high prevalence rate of *A. baumannii* in hospitalized and immunocompromised patients. Moreover, improper use of antibiotics has led to the emergence of extensive drug-resistant strains that urgently require alternative strategies to control this superbug. Unfortunately, the availability of a licensed vaccine against *A. baumannii* infections is still challenged by the vast diversity among *A. baumannii* strains. Here, we report the development of a novel pentavalent vaccine candidate composed of two recombinant proteins (Wza and YiaD) and a pool of capsular polysaccharides isolated from 3 clinical isolates. We tested this new vaccine *in vivo* in a mouse model of peritonitis against the standard strain ATCC 19606 in addition to 3 clinical isolates of *A. baumannii*. Immunization with this vaccine completely protected the challenged mice with 100% survival rate in the case of all the tested bacteria. Further clinical studies are urgently needed to evaluate the efficacy and safety of this proprietary vaccine to protect patients from *A. baumannii* lethal infections.

**Key points:**

*• Recombinant proteins pool (Wza and YiaD) immunization led to a synergistic immune response.*

*• Capsular polysaccharides pool induced up to 90% protection of tested clinical isolates.*

*• The pentavalent pool showed superiority with 100% survival of immunized mice.*

**Supplementary Information:**

The online version contains supplementary material available at 10.1007/s00253-022-12231-3.

## Introduction

*Acinetobacter baumannii* is a non-motile Gram-negative coccobacillus (Elhosseiny and Attia 2018). Although most *Acinetobacter* species are considered non-pathogenic environmental organisms (Geisinger et al. 2019; Wong et al. 2017), *A. baumannii* is considered one of the most dangerous opportunistic pathogens. It affects immunocompromised and hospitalized patients mainly (Elhosseiny and Attia 2018; Kroger et al. 2016), where long intensive care unit (ICU) stay has been recognized as the number one predisposing factor for *A. baumannii* infections (Ayoub Moubareck and Hammoudi Halat 2020; Kroger and Kary 2016; Lee et al. 2017; McConnell et al. 2013). This serious pathogen could cause multiple infections including pulmonary, urinary tract infections, osteomyelitis, traumatic or post-surgical wound infections, bacteremia, and post-neurosurgical meningitis (Ayoub Moubareck and Hammoudi Halat 2020; Gellings et al. 2020; Piperaki et al. 2019). The mortality rate of *A. baumannii*-associated bacteremia and pneumonia is 60% and 70%, respectively (Gellings and Wilkins 2020). *A. baumannii* is equipped with an array of virulence determinants that enhance its ability to evade host immune response and survive in hospital environments (Cerqueira and Peleg 2011; Dijkshoorn et al. 2007; Harding et al. 2018). Outer membrane proteins (OMPs), cell envelope-associated factors, biofilm formation, secretory systems, quorum sensing, and micronutrient acquisition systems have been recognized as the major virulence factors that aid *A. baumannii* to resist antibiotics, invade host cell, and finally, trigger apoptosis (Morris et al. 2019; Skariyachan et al. 2019).

*A. baumannii* has also developed remarkable antibiotic resistance mechanisms including: upregulated multidrug efflux pumps, enzymatic modification of antibiotics, target gene mutation, and altered outer membrane permeability (Ayoub Moubareck and Hammoudi Halat 2020; Nowak and Paluchowska 2016; Piperaki and Tzouvelekis 2019). The combined effect of those mechanisms has led to the emergence of multi drug resistant (MDR) and even extensive drug-resistant (XDR) strains of *A. baumannii* (Gellings and Wilkins 2020; Kroger and Kary 2016; Martins et al. 2013). Carbapenems were initially considered for treatment of MDR *A. baumannii*; however, colistin and tigecycline were then used to treat carbapenem-resistant strains ( Benmahmod et al. 2019; Piperaki and Tzouvelekis 2019; Skariyachan and Taskeen 2019). Unfortunately, many studies have recently revealed the emergence of colistin- and/or tigecycline-resistant *A. baumannii* clinical isolates, rendering the last resort antibiotic treatment ineffective (Sun et al. 2018). The World Health Organization has recognized MDR and XDR *A. baumannii* as the number one critical priority pathogen that urgently requires new therapeutics, where concerns are growing continuously about the fact that hospital-acquired *A. baumannii* infections will soon be untreatable (Morris and Dexter 2019; Shlaes and Bradford 2018). This stressful combat strongly encourages the medical community to focus on primitively preventing infection through different vaccination strategies. As far as we know, there is still no licensed vaccine against *A. baumannii* regardless of the major research effort that has suggested multiple vaccine candidates that have been proved effective at some levels in pre-clinical trials [reviewed in (Gellings and Wilkins 2020)]. However, the major challenge in development of an effective vaccine remains to be the vast diversity among *A. baumannii* clinical isolates (Singh et al. 2018).

Active immunization utilizes different antigens, such as whole cell, pure proteins, or capsular polysaccharides as vaccine candidates (Ahmad et al. 2019, 2016; Hosseingholi et al. 2014). Incomplete inactivation of the bacterial cell and the presence of pyrogenic endotoxins are the major drawbacks of whole cell-based immunization, which might initiate infection or unfavorable immune response, respectively (Moriel et al. 2013). Protein-based bacterial components have been extensively evaluated as vaccine candidates, where the use of *in silico* computational mapping techniques has massively contributed to the identification of novel potent *A. baumannii* epitopes (Ahmad and Ranaghan 2019; Hosseingholi and Rasooli 2014). These proteins include phospholipase D, outer membrane protein BamA and FilF, vesicle’s outer membrane proteins FKIB and Ompp1, outer membrane protein nuclease NucAb, Surface Loops of ZnuD, the functional exposed amino acid BauA, and outer membrane proteins (OmpA, OmpW, OmpK, and Omp22) that induce specific, desirable, and broad-spectrum humoral and cellular immunity (Ahmad and Tawfik 2016; Garcia-Quintanilla et al. 2013; Gellings and Wilkins 2020; Qamsari et al. 2020). However, the efficacy of these proteins still needs further evaluation against different *A. baumannii* clinical isolates. Capsular polysaccharides have revealed promising protection levels against certain standard strains of *A. baumannii* in pre-clinical trials (Hosseingholi and Rasooli 2014). Regarding their composition, capsules are formed of repeated linear or branched polysaccharide units containing unique glycans with atypical acetylation sites or may be exclusively decorated with amino sugars (Kasimova et al. 2017). Therefore, the high variations in composition of capsular polysaccharides limit their effectiveness as vaccine candidates against various *A. baumannii* clinical isolates (Yang et al. 2017). Nevertheless, the use of a capsule-based vaccine is generally associated with short-term immune protection as it relays primarily on T cell independent immune stimulation (Russo et al. 2013).

In this study, we have evaluated the individual and pooled (pentavalent) protective effects of two newly employed recombinant proteins (YiaD and Wza) and capsular polysaccharides isolated from different clinical isolates as vaccine candidates against *A. baumannii* infections using the *in vivo* mouse model of peritonitis.

## Materials and methods

### Bacterial strains and isolates

#### Standard strains

*E. coli* BL-21(DE3)pLysS and Top10 standard strains were purchased from Novagen and Invitrogen (USA), respectively. The standard ATCC 19606 was used as the standard strain throughout our study.

#### Clinical isolates and their identification

One hundred *A. baumannii* clinical isolates were recovered from patient specimens in Mansoura University Hospitals between April 2019 and November 2019. Isolates were recovered from blood (39 samples), sputum (35 samples), urine (16 samples), and wound (10 samples). They were identified as *A. baumannii* according to standard microbiological techniques, including colony morphology, Gram stain, biochemical reactions, and molecular-based methods (PCR detection of both *gltA* and *bla*_*OXA-51-like*_ genes) (Benmahmod et al. 2019; Said et al. 2018).

### Recombinant proteins

#### Cloning, expression, and purification of Wza and YiaD

Genomic DNA of *A. baumannii* standard ATCC 19606 strain was extracted and used as template for PCR. *Wza* (OmpA family lipoprotein) and *YiaD* (outer membrane protein; involved in capsule export) genes were cloned and expressed individually using standard protocols. The coding sequences for *Wza* and *YiaD* genes were amplified using custom-designed primer pairs (Table 1). The designed primers were tagged with restriction sites of *Bam*HI and *Eco*RI (Thermo Fisher Scientific, USA), and designed to allow the amplified coding sequence to fit in-frame with the expression vector pRSET-B (Invitrogen, USA). The pRSET-B/*Wza* and pRSET-B/*YiaD* recombinant vectors were transformed individually into chemically competent *E. coli* Top10 and plated on LB/ampicillin (100 µg/ml) agar plates. Recombinant vectors were extracted from the transformed colonies and tested by double-digestion/gel-electrophoresis. pRSET-B/*Wza* and pRSET-B/*YiaD* recombinant vectors were transformed into chemically competent *E. coli* BL-21(DE3)pLysS and then plated on LB/ampicillin agar plates. Single transformed colony was transferred into 20 ml of LB/ampicillin and incubated at 37 °C overnight with shaking at 150 rpm. Finally, LB broth (500 ml) was inoculated with the overnight culture and incubated at 37 °C till OD_600_ = 0.1; then further incubated at 30 °C and 37 °C for Wza and YiaD, respectively, with 200 rpm shaking till OD_600_ = 0.4–0.6. Isopropyl-β-D-thiogalactopyranoside (IPTG) (Sigma-Aldrich, USA) was added at final concentration of 1 mM to induce protein expression, culture was further allowed to grow under the same conditions for 6 h. Cells were then harvested and transferred to the two-step purification stage. Firstly, inclusion bodies purification protocol was applied to both proteins according to the previously described protocol (Almansoor 2017). Purified inclusion bodies were then solubilized according to GE Healthcare Bio-Sciences AB 1999. The obtained solubilized proteins were then purified using Ni^+2^ Sepharose 6 Fast Flow packed column (GE Healthcare) following the manufacturer’s directions using 25 mM and 250 mM imidazole in washing and elution steps, respectively. To identify and confirm the purity of obtained recombinant proteins, 20-µl samples were screened on SDS-PAGE and western blot using anti-histidine tagged antibodies (Mellick and Rodgers 2007), to detect the expected bands of Wza and YiaD at 28.89 and 22.49 kDa, respectively.

**Table 1:** List of Primers used in this study

Target Gene		Primer Sequence (5`→ 3`)	Annealing Temp (°C)	Product Size (bp)	Accession Number
*Wza*	Fw	GCGGATCCGCCTAGTGAGGGTGTTTATAAAACG	55	789	AP022836
	*Rv*	GCGAATTCGCTTGCACTTGCAGAGTTTGG			REGION: 3868441–3869541
*YiaD*	Fw	GCGGATCCGCGTGCATTAGTTATTTCAACAGTG	55	630	AP022836
	Rv	GCGAATTCGATTTCTACACGGCGGTTTTG			REGION: 3072929–3073582

Restriction enzymes sites engineered into each primer are underlined. Sites are GGATCC for *Bam*H1 andGAATTC for *Eco*R1

*Fw* forward primer, *Rv *reverse primer

#### Immunization of mice with either Wza or YiaD recombinant proteins

Purified recombinant proteins were used to immunize female BALB/c mice (20–25 gm weight) as described previously with minor modifications (Girgis et al. 2020). In brief, each mouse was injected subcutaneously (SC) with a mixture of 300 µg protein and 2 mg aluminum hydroxide [Alum (Sigma-Aldrich Co., USA)] as adjuvant, in 200 µl saline. Mice received the same dose weekly for three consecutive weeks as booster doses. A control group was immunized with alum only. Blood samples were collected at day zero (as a negative control), 14, 21, and 28, to determine the antibody titer in the collected sera using enzyme-linked immuno-sorbent assay (ELISA).

#### Recombinant proteins pool

A pool of equimolar concentrations of both Wza and YiaD was used to immunize female BALB/c mice. Each mouse received a total of 600 µg proteins (300 µg of each recombinant protein) and 2 mg alum per dose at the same time intervals as mentioned before. Immune sera formed against the pool of proteins were then isolated at day 28 from the start of immunization protocol to screen their cross-reactivity with the clinical *A. baumannii* isolates using ELISA.

### Capsular polysaccharides

#### Extraction and quantification of capsular polysaccharides

Capsular polysaccharides of the standard strain ATCC 19606 in addition to selected clinical isolates (isolates No. 51, 63, 76, 79, and 100) were extracted as previously described (Tipton and Rather 2019). Briefly, bacteria were streaked on LB agar plates and incubated at 37 °C for about 18 h. Cells were then scraped and suspended in nutrient broth to optical density (OD_600_) about 0.65. Bacterial cells were harvested from 1 ml of suspension via centrifugation. The pellet was re-suspended in 200 μl lysis buffer (2.4 ml 1 M Tris–HCL; pH 8, 0.0002 g CaCl_2_, 0.081 g MgCl_2_ and 3 mg/ml Lysozyme). Suspensions were then incubated at 37 °C for 60 min followed by vortex and three consecutive cycle extractions (freeze/thaw cycles) at − 80 °C and 37 °C. Following that, 1 μl of both DNase and RNase (20 μg/ml each) were added to extracts and incubated at 37 °C for 30 min; then 10 μl of 10% SDS were added and incubated at 37 °C for 30 min. Extracts were boiled in water bath at 100 °C for 10 min and allowed to cool at room temperature. At this point, 150 μl of lysis buffer containing proteinase K (2 mg/ml) were added to extracts and incubated for 60 min at 60 °C; then extracts were centrifuged at room temperature for 2 min at maximum speed. Supernatants were transferred to clean microcentrifuge tubes, to which 400 μl of cold 75% ethanol was added, mixed by inverting the tubes, and incubated at − 20 °C for overnight precipitation. Finally, tubes were centrifuged at 4 °C for 30 min at maximum speed and supernatants were aspirated. Once tubes were dried of residual solution, capsule pellets were suspended in Tris-buffered saline (TBS; 10 mM Tris, 140 mM NaCl, pH 7.4).

Capsular polysaccharides were quantified through colorimetric analysis according to the previously utilized protocol (Brimacombe and Beatty 2013) utilizing a standard calibration curve (Fig. S1). In brief, 200 μl of each extract was swirled with 200 μl of 5% phenol and 1 ml of 93% sulfuric acid. Color was allowed to develop at room temperature for 10 min with subtle swirling every 2–3 min. Optical density (OD_490_) was then measured, and the concentration of capsular polysaccharides was calculated from the standard curve obtained using serial dilutions of carbohydrate stock (1:1 mixture of 0.5 mg/ml each of sucrose and fructose).

#### Immunization of mice with capsular polysaccharides

Capsular polysaccharides were used to immunize mice as described previously (Kurbatova et al. 2017) with some modifications. Female BALB/c mice (20–25 gm weight) were SC immunized with capsular polysaccharides supplemented with alum. Mice were dosed 4 times at days 0, 7, 14, and 21, where each mouse received a mixture of 10 µg capsular polysaccharides and 2 mg alum in 200 µl saline per dose. A control group was immunized with alum only. Blood samples were collected at day zero (as a negative control), 14, 21, and 28, to determine the antibody titer in the collected sera using ELISA.

#### Capsular polysaccharides pool

A pool of equimolar concentrations of the capsular polysaccharides extracted from 3 clinical isolates (No. 51, 63, and 76) was used to immunize female BALB/c mice. Where each mouse received a total of 30 µg capsular polysaccharides (10 µg of capsular polysaccharides of each clinical isolate) and 2 mg alum per dose, at the same time intervals as described previously. Immune sera formed against the pool were isolated at day 28 from the start of immunization protocol to screen their cross-reactivity with all 100 clinical *A. baumannii* isolates using ELISA.

### Pentavalent pool

A pentavalent pool combining both recombinant proteins (Wza and YiaD) and capsular polysaccharides of three clinical *A. baumannii* isolates was used to immunize female BALB/c mice groups (*n* = 10/group). Each mouse received a total of 600 µg proteins (300 µg of each recombinant protein) and 30 µg capsular polysaccharides (10 µg of capsular polysaccharides of each clinical isolate) in addition to 2 mg alum per dose, by the same time intervals as mentioned before. Immune sera formed against the pentavalent pool were then used to screen their cross-reactivity with *A. baumannii* clinical isolates using ELISA.

### ELISA-based assessment of serum antibody titer following immunization

Serum antibody titer assessments were performed according to Huang et al. (2016) in the case of recombinant proteins, and according to Kurbatova et al. (2017) in the case of capsular polysaccharides, with minor modifications. Microtitre plates (Maxisorp, Nunc, Sigma-Aldrich, USA) were coated with 100 µl/well of 10 µg/ml of either recombinant proteins (Wza and YiaD) or capsular polysaccharides in coating buffer (15 mM Na_2_CO_3_, 35 mM NaHCO_3_, pH 9.6) and incubated at 4 °C overnight. Residual protein-binding sites were then blocked with 250 µl/well of 1% (w/v) bovine serum albumin (Sigma-Aldrich, USA) in TBS buffer at room temperature. After 2 h, plates were washed three times with 250 µl/well of wash buffer (TBS supplemented with 5 mM CaCl_2_ and 0.05% Tween 20). Serum samples were serially diluted in TBS with 2 mM CaCl_2_, added to plates (100 µl/well), and incubated at room temperature for 90 min. Plates were washed once more and bound mouse immunoglobulin (IgG) was determined using alkaline phosphatase-conjugated goat anti-mouse antibody and the chromogenic substrate para-nitrophenyl phosphate (Sigma-Aldrich, USA). Finally, absorbance at 405 nm was measured using Microtitre plate reader (Biotek instruments inc., USA).

### Screening of cross-reactivity of different immune sera with clinical *A. baumannii* isolates

ELISA technique was used to determine the cross-reactivity of the immune serum developed against each immunogen (recombinant proteins, capsular polysaccharides, or the pentavalent pool) with *A. baumannii* clinical isolates. ELISA assays were carried out according to the previously described protocol (Kohl and Ascoli 2017; Bidmos et al., 2018) with minor modifications, where *A. baumannii* isolates were cultured at 37 °C with shaking at 2000 rpm in LB broth media overnight. The pellets were obtained by centrifugation at 4000 rpm for 10 min and washed 3 times with TBS. Bacteria were then fixed in 0.5% (v/v) formaldehyde in TBS at room temperature for an hour. Following the fixation, cells were washed three times with TBS and then re-suspended in coating buffer. Wells of Microtitre plate were coated with the formalin-fixed *A. baumannii* suspensions of OD_600_ = 0.5 in 100 μl aliquots in coating buffer and incubated overnight at 4 °C. Residual protein-binding sites were then blocked with 250 µl/well of 1% (w/v) bovine serum albumin in TBS buffer at room temperature. After 2 h, plates were washed three times with 250 µl/well of wash buffer. Immune serum collected from mice after one week of the last immunization booster dose was diluted to 1/5000 ratio using TBS, added to plates (100 µl/well) and incubated at room temperature for 90 min. Plates were washed again and bound mouse immunoglobulins were determined using alkaline phosphatase-conjugated goat anti-mouse antibody and the chromogenic substrate para-nitrophenyl phosphate. Absorbance at 405 nm was measured using Microtitre plate reader.

### Bacterial challenge and survival experiments

Firstly, the challenging dose of ATCC 19606 standard strain was determined through injecting female BALB/c mice groups (*n* = 10/group) intra-peritoneally with different doses of bacteria in LB broth combined with 10% porcine mucin type II (Sigma-Aldrich, USA) as adjuvant in 200 µl inocula. Porcine mucin has been formerly proved to augment the infectivity of *A. baumannii* and other bacteria in various experimental models, thus allowing us to use smaller inocula (McConnell et al. 2011a, b; McConnell et al. 2011a, b). Each group received a different dose including 1 × 10^7^, 1 × 10^8^, 1 × 10^9^ and finally 5 × 10^8^ colony forming unit (CFU), based on a previously determined relation curve between optical density (OD_600_) and CFUs. The control group received porcine mucin only. All mice were monitored for clinical signs and disease progression for 7 days.

Immunized, using the same protocol as previously described, and control non-immunized mice (*n* = 10/group) were then challenged, 1 week after the last immunization, intra-peritoneally with 200 µl inocula (100 µl of 5 × 10^8^ CFU of *A. baumannii* and 100 µl of 10% porcine mucin). Viable counts were performed to determine the actual numbers of CFUs in the injected inocula. Mice were monitored for clinical signs and disease progression for 7 days (Huang et al. 2014).

### Evaluation of bioburden in mice tissues

Tissue bacterial burdens were evaluated as described previously (Ainsworth et al. 2017) with minor modifications. Briefly, spleens, livers, and kidneys from both immunized and control non-immunized groups were collected at 5 different time intervals including 4, 8, 12, 24, and 48 h post *A. baumannii* challenge. Tissues were collected from three mice at each time interval in each group and homogenized separately in 2 ml sterile saline. Following that, tenfold serial dilutions were plated on nutrient agar plates using surface-drop viable count technique to estimate bacterial burdens and finally normalized by the tissue weight. Negative control mice tissues were also collected and plated to exclude false results.

### Histopathological examination of mice tissues

Livers were collected from immunized and non-immunized control mice at 8, 24, and 48 h post-challenge with *A. baumannii* isolates, as well as negative control uninfected mice. Tissues were collected from three mice at each time interval in each group, suspended in 2 mL of 10% formalin, fixed in wax of paraffin, sectioned then stained with hematoxylin–eosin (H&E) for histopathological observation. Three sections from each mouse liver tissue were examined for quantitation of leukocyte infiltration rates. Liver sections were examined microscopically in a blind manner and scored by a veterinary pathologist according to the severity and extent of vascular congestion, hepatocellular degeneration, hepatocellular necrosis, polymorphonuclear inflammation, and leukocytes infiltration (Ainsworth et al. 2017).

### Statistical analysis

Graph Pad Prism software package (version 6.01) was used to statistically analyze survival experiments using Mantel-Cox log-rank test; with *n* = 10/group, while multiple *t*-test was used in the case of bioburden assays where statistical significance was calculated using Holm-Sidak method, with alpha = 5.000%. Unpaired *t*-test was used to analyze histological variation levels in liver tissues. All results were estimated as the mean of three separate experiments and considered significant when p < 0.05.

## Results

### Recombinant proteins

#### Cloning of *Wza* and *YiaD* coding sequences into pRSET-B expression vector

**Fig. 1:**
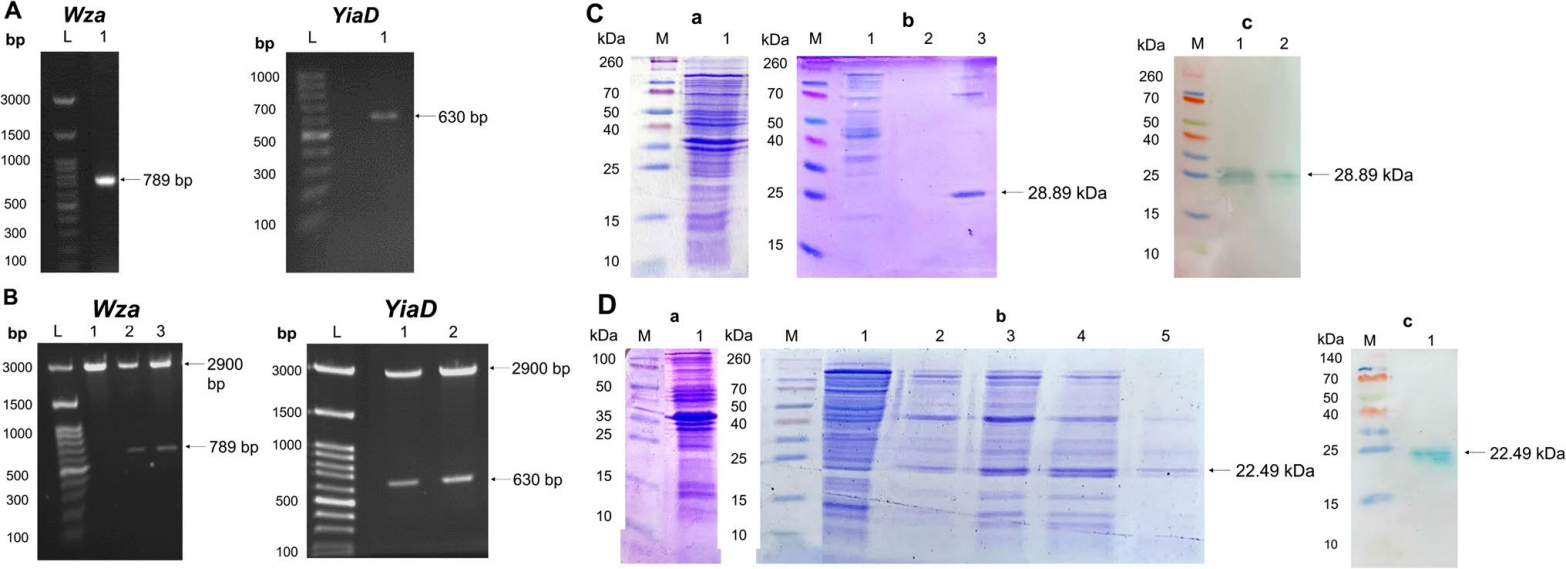
PCR amplification of the coding sequences of both *wza* and *YiaD* (**A**), construction of recombinant expression vectors pRSET-B/*wza* and pRSET-B/*YiaD* (**B**), and identification and purification of Wza (**C**) and YiaD (**D**) recombinant proteins. In **A**, genomic DNA of the standard strain was used as a template for the amplification of target genes. Lanes L: 100 bp DNA ladder H3 RTU; lanes 1: PCR amplification of the 789 bp, and 630 bp coding sequences of *Wza* and *YiaD*, respectively. In **B**, double digestion of *Wza* (lanes 2 and 3) and *YiaD* (lanes 1 and 2) constructs in pRSET-B (2900 bp) with *Bam*HI and *Eco*RI, confirmed the presence of *Wza* (789 bp) and *YiaD* (630 bp) inserts; lanes L: 100 bp DNA ladder H3 RTU. **C** Identification of Wza (28.89 kDa), **C** (a): Wza protein before purification on 15% SDS-PAGE; lane M: Spectra™ multicolor broad range protein marker. **C** (b): purified Wza protein (Ni^+2^ Sepharose column); lane 1: column wash, lanes 2 and 3: different column elution fractions, lane 3: purified band of Wza. **C** (c): Western blot analysis of Wza (anti-histidine tagged monoclonal antibodies) showing Wza band at approximately 28.89 kDa. **D** identification of YiaD (22.49 kDa), **D** (a): YiaD before purification on 15% SDS-PAGE; lane M: Spectra™ multicolor broad range protein marker. **D** (b): purified YiaD protein (Ni^+2^ Sepharose column); lane 1: column wash, lanes 2–5: different column elution fractions, lane 5: purified band of YiaD. **D** (c): Western blot analysis of YiaD (anti-histidine tagged monoclonal antibodies) showing YiaD band at approximately 22.49 kDa

Amplicons of 789 bp and 630 bp were obtained following PCR amplification of *Wza* and *YiaD* coding sequences, respectively (Fig. 1A) utilizing genomic DNA of standard *A. baumannii* strain (ATCC 19606) as a template. Successful cloning of coding sequences in the expression vector pRSET-B was confirmed by detecting restriction analysis products of 789 bp or 630 bp and 2900 bp, corresponding to *Wza* or *YiaD* and vector, respectively (Fig. 1B).

#### Expression and purification of Wza and YiaD proteins

Optimal expression of recombinant Wza and YiaD was observed 6 h following induction with IPTG in *E. coli* BL-21(DE3)pLysS. Following purification, protein bands of 28.89 and 22.49 kDa corresponding to the expected sizes of Wza (Fig. 1C) and YiaD (Fig. 1D), respectively, were observed in SDS-PAGE. Bands of the purified proteins were identified by anti-histidine tagged antibodies following Western blot (Fig. 1C and D).

#### Immunization with either Wza or YiaD recombinant proteins

Purified recombinant proteins Wza or YiaD were used to immunize groups of BALB/c mice (*n* = 3/group). Collected sera at different time intervals from both immunized and control mice that received alum only were used to assess the antibody titer following immunization using ELISA. Immunized mice showed a robust increase in antibody titer following each booster dose of immunization, while the control group showed no immune response against Wza or YiaD (Fig. S2A and B, respectively).

For all survival assays, the challenging dose of standard *A. baumannii* strain (ATCC 19606) was firstly determined. Infectious doses of 1 × 10^7^ and 1 × 10^8^ CFU/mouse of the standard strain were associated with survival rates higher than 40% of the challenged mice. Increasing the infectious dose to 5 × 10^8^ CFU/mouse resulted in a reduction of survival to 20%. A dose of 1 × 10^9^ CFU/mouse was associated with no survivals among all the challenged mice. Survival rate was 100% among control mice that received mucin only (Fig. S3). Therefore, in subsequent survival experiments, mice were challenged with 5 × 10^8^ CFU/mouse corresponding to LD_80_.

**Fig. 2:**
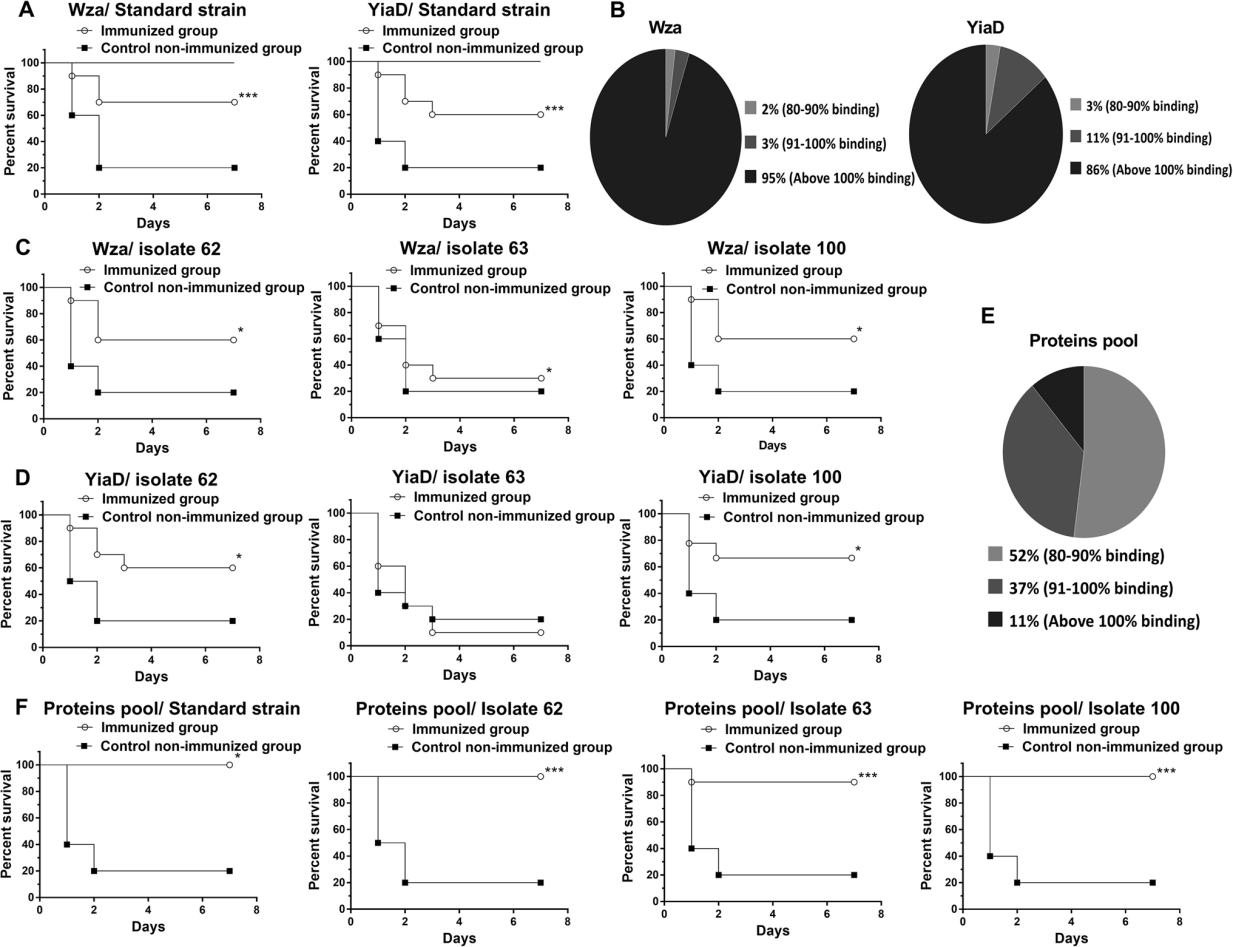
Survival experiments of immunized mice with either Wza, YiaD recombinant proteins, or their pool together against *A. baumannii* challenge, and Cross-reactivity of immune sera developed against either Wza, YiaD recombinant proteins, or their pool together with 100 clinical isolates (**B** and **D**). **A** Immunization with Wza alone significantly protected the challenged mice with 70% survival rate against the standard strain, while YiaD immunization has led to 60% survival rate against the same standard strain. **B** Different levels of cross-reactivity between immune sera formed against Wza or YiaD and tested clinical isolates. **C** Wza immunization significantly protected the challenged mice with survival rates up to 60% against clinical isolates No. 62 and No. 100, while no protection was observed with isolate No. 63 with 30% survival rate. **D** Challenging YiaD immunized mice with clinical isolates No. 62 and 100 led to 60% and 70% survival rates, respectively; however, survival following the challenge with clinical isolate No. 63 was only 10%. **E** Forty-eight percent of the clinical isolates showed more than 90% binding with the immune serum of mice immunized with the proteins pool, while the remaining 52% of isolates showed binding ranging from 80 to 90%. **F** Recombinant proteins pool immunization resulted in complete protection of immunized mice (100% survival rate) against each of the standard strain, clinical isolates No. 62 and No. 100, while a 90% survival rate was observed with isolate No. 63. Control non-immunized mice groups showed 20% survival rate in all survival experiments. * *P* < 0.05, ** *P* < 0.01 and *** *P* < 0.001

Immunization of mice groups (*n* = 10/group) with either Wza or YiaD significantly protected mice against the challenging dose (LD_80_) of the standard ATCC 19606 strain, where Wza or YiaD immunization significantly increased survival to 70% or 60%, respectively, in comparison to 20% in the case of the non-immunized control group (Fig. 2A).

To further evaluate the efficacy of the two recombinant proteins as vaccine candidates against *A. baumannii* clinical isolates, the ELISA technique was used to determine the cross-reactivity between these clinical isolates and the isolated immune sera containing antibodies formed against each recombinant protein. In the case of Wza immunization, only 2% of isolates showed less than 90% binding (100% in the case of the standard strain) with the antibodies formed against Wza immunogen, while YiaD immunization induced formation of antibodies in immune sera that showed more than 90% binding affinity (100% in the case of the standard strain) with 97% of tested clinical *A. baumannii* isolates (Fig. 2B).

To confirm these results, each recombinant protein was used to immunize three groups of female BALB/c mice (*n* = 10/group). Immunized groups were challenged with LD_80_ of clinical *A. baumannii* isolates (No. 62, 63, and 100) at day 28 from the immunization protocol beginning. Immunization of mice with Wza recombinant protein protected the challenged mice against clinical isolates No. 62 and No. 100 with 60% survival rate, while failed to protect mice against isolate No. 63 with only 30% survival rate (Fig. 2C). Challenging YiaD immunized mice with clinical isolates No. 62 and No. 100 lead to 60% and 70% survival rates, respectively; however, survival following the challenge with clinical isolate No. 63 was only 10% (Fig. 2D).

#### Recombinant proteins pool

The two recombinant proteins (Wza and YiaD) were pooled together, in equimolar concentrations, and used as one immunogen. Group of three female BALB/c mice were immunized with the recombinant proteins pool, immune sera were collected at day 28 and used to screen their cross-reactivity with all 100 clinical *A. baumannii* isolates. ELISA results revealed that 48% of isolates exceeded 90% binding with immune serum (100% in the case of the standard strain), while the remaining 52% of tested clinical isolates showed binding levels of 80% to 90% (Fig. 2E).

Following that, the recombinant proteins pool was used to immunize four groups of mice (*n* = 10/group). Immunization with the recombinant proteins pool completely protected the challenged mice with 100% survival rate in the case of the standard strain, clinical isolates No. 62 and No. 100. In addition, a 90% survival was observed upon challenging proteins pool immunized mice with the clinical isolate No. 63 (Fig. 2F).

### Capsular polysaccharides

#### Capsular polysaccharides of the standard ATCC 19606 strain

**Fig. 3:**
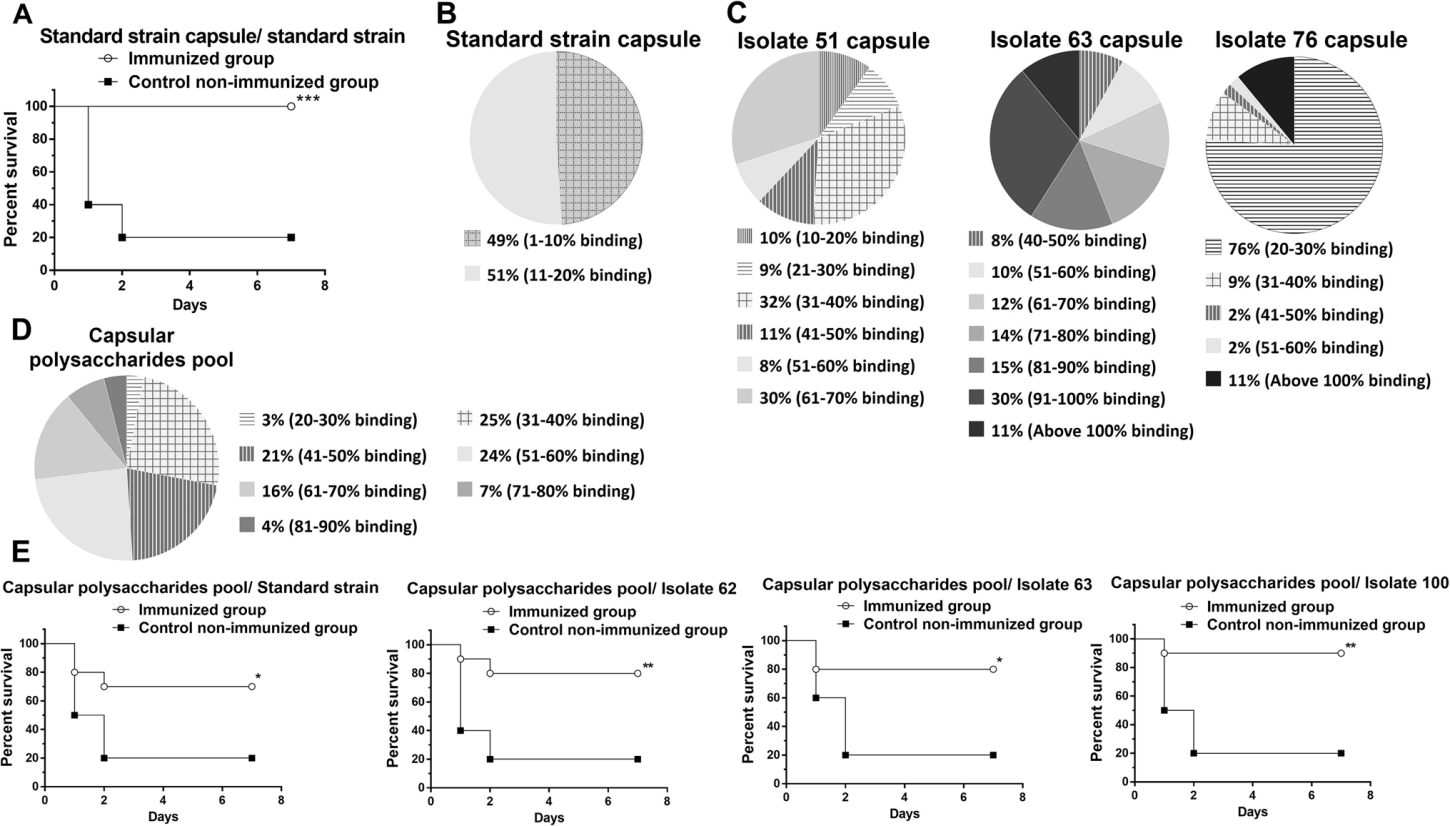
Survival experiments of immunized mice with capsular polysaccharides against *A. baumannii* challenge (**A** and **E**) and cross-reactivity of immune sera developed against different capsular polysaccharides with 100 clinical isolates (**B**–**D**). **A** Immunization with capsular polysaccharides of the standard strain completely protected the challenged mice with 100% survival rate against LD_80_ of the standard strain. **B** Minimal cross-reactivity levels between immune serum developed against the capsular polysaccharides of the standard strain and all tested clinical isolates. **C** Variable levels of cross-reactivity with all 100 tested clinical isolates following immunization with capsular polysaccharides isolated from three different clinical isolates, namely No. 51, 63, and 76. **D** Cross-reactivity levels in the case of immunization with the capsular polysaccharides pool, where more than 50% antibody binding was observed with 51 clinical isolates. **E** Immunization with the capsular polysaccharides pool protected the challenged mice with 70% survival rate against the standard strain, 80% survival rates against both clinical isolates No. 62 and 63, while a 90% survival rate was observed in the case of isolate No. 100. Control non-immunized mice groups showed 20% survival rate in all survival experiments. * *P* < 0.05, ** *P* < 0.01 and *** *P* < 0.001

Capsular polysaccharides of *A. baumannii* ATCC 19606 standard strain were extracted, quantified (Fig. S1), and used to immunize a group of 3 BALB/c mice, where ELISA results revealed a significant increase in antibody titer in immune sera isolated from immunized mice upon injecting booster immunization doses (Fig. S2C). Immunization with the extracted capsular polysaccharides completely protected the challenged mice (*n* = 10) with 100% survival rate against the standard strain (Fig. 3A). However, ELISA results showed minor antibody cross-reactivity (not exceeding 20% binding) with all tested clinical isolates (Fig. 3B).

#### Capsular polysaccharides pool

Based on previous ELISA results, the capsular polysaccharides of five *A. baumannii* clinical isolates (isolates No. 51, 63, 76, 79, and 100) were extracted and quantified. Each of these capsular polysaccharides were used to immunize mice separately (*n* = 3/group), immune sera were then collected and used to screen their cross-reactivity with the clinical *A. baumannii* isolates. ELISA results revealed that immune sera formed against capsular polysaccharides of isolates No. 79 and 100 showed no cross-reactivity with all the other tested clinical isolates. However, variable cross-reactivity levels were detected in the case of immunization with capsular polysaccharides of isolates No. 51, 63, or 76 as shown in Fig. 3C. Thus, a pool of equimolar concentrations of the capsular polysaccharides extracted from these clinical isolates (No. 51, 63, and 76) was used to immunize a group of three mice. Immune serum formed against the polysaccharides pool was then used, through ELISA, to screen its cross-reactivity with the clinical *A. baumannii* isolates. ELISA results revealed 11 clinical isolates had more than 70% binding (100% binding in the case of isolate No. 51), 86% of isolates ranged from 70 to 30% binding, and eventually, only 3 isolates had less than 30% binding (Fig. 3D). Upon challenging immunized mice with the capsular polysaccharides pool, survival rates were 70%, 80%, 80% and 90% for the standard strain, clinical isolates No. 62, 63, and 100 (Fig. 3E), respectively.

### Pentavalent pool

**Fig. 4:**
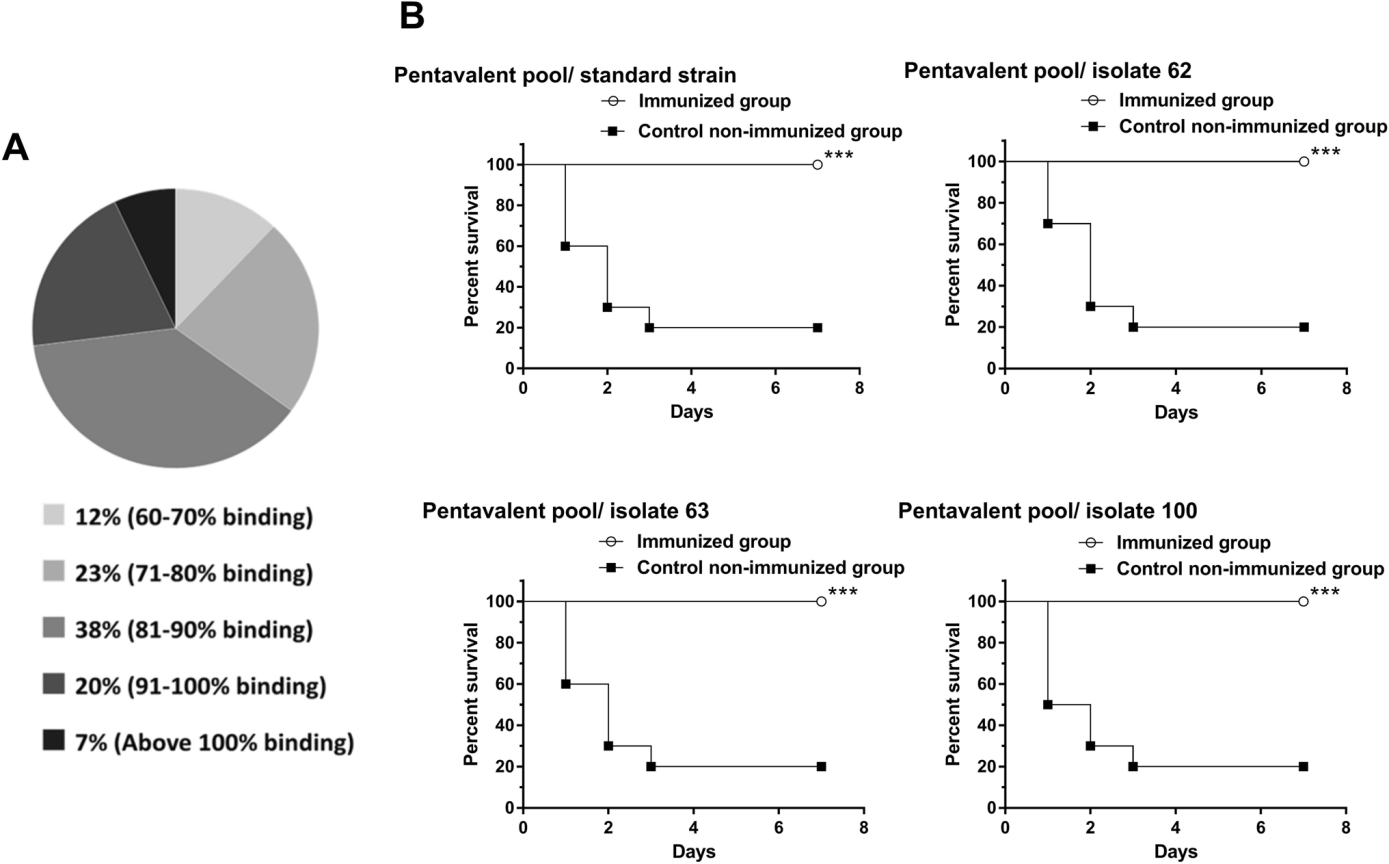
Cross-reactivity of the immune serum developed against the pentavalent pool with 100 clinical isolates (A) and survival experiments of immunized mice with the pentavalent pool against *A. baumannii* challenge (**B**). **A** All tested isolates showed higher than 60% binding with the immune serum of immunized mice with the pentavalent pool. **B** Immunization with pentavalent pool completely protected the challenged mice with 100% survival rate against all tested bacteria including the standard strain and three clinical isolates (No. 62, 63, and 100). Control non-immunized mice groups showed 20% survival rate in all survival experiments. * *P* < 0.05, ** *P* < 0.01, and *** *P* < 0.001

Both recombinant proteins and capsular polysaccharides pools were mixed together giving a pentavalent vaccine candidate that was then evaluated in protection against *A. baumannii* challenge. The new vaccine candidate was evaluated *in vivo* in mice against the standard strain, in addition to three clinical isolates of *A. baumannii* (No. 62, 63, and 100). Immune serum from immunized mice with the pentavalent pool showed higher binding than that of the standard strain (100% binding) with seven tested isolates, whereas 71–100% binding was observed with 81% of tested clinical isolates, and 60–70% binding was detected with 12% of tested isolates (Fig. 4A). Immunization with the pentavalent pool completely protected the challenged mice with 100% survival in case of all tested bacterial challenges (Fig. 4B).

### Evaluation of bioburden in mice tissues

**Fig. 5:**
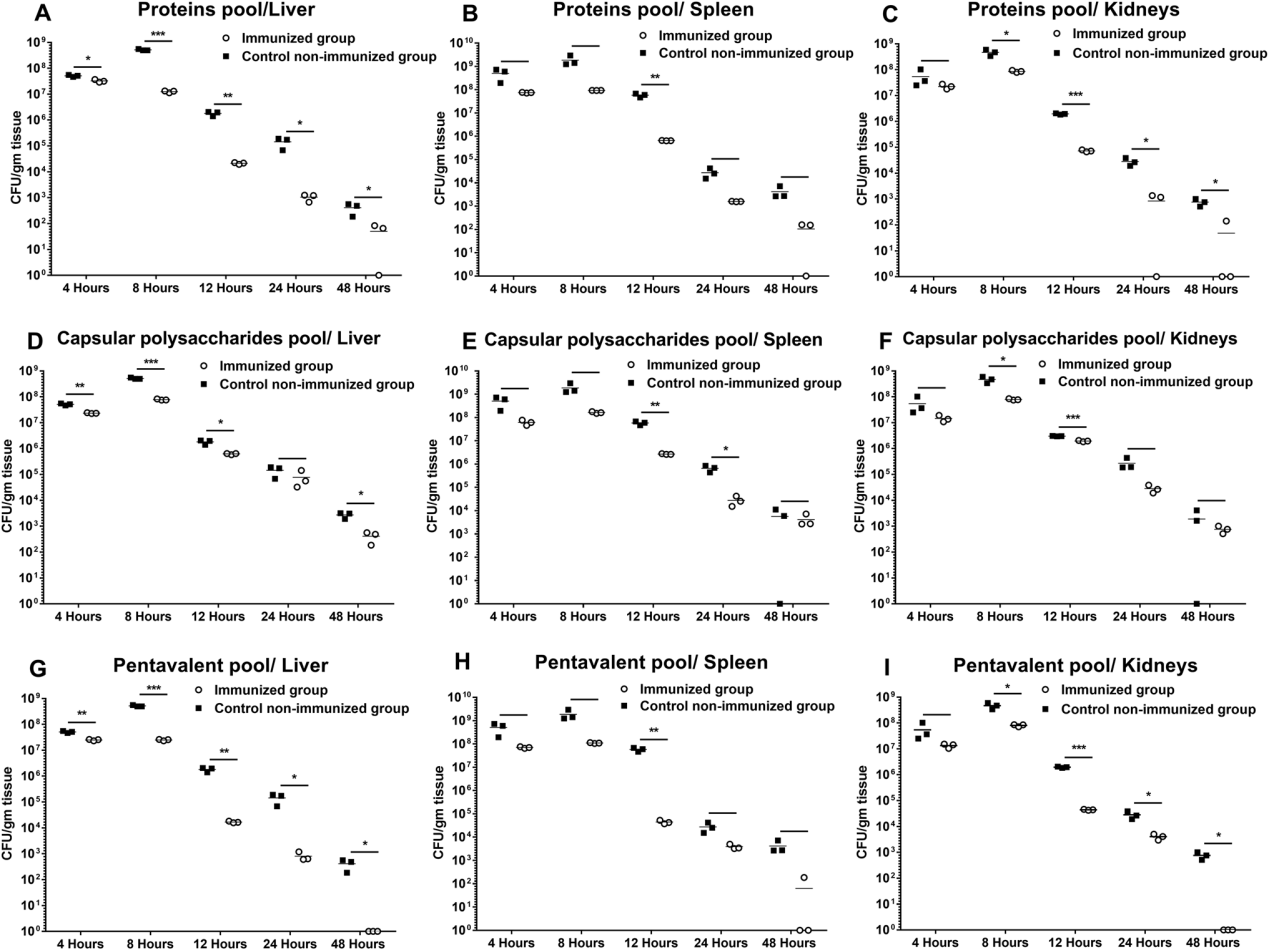
*A. baumannii* bioburdens in the liver, spleen, and kidneys of immunized mice with the recombinant proteins pool (**A**–**C**), the capsular polysaccharides pool (**D**–**F**), and the pentavalent pool (**G**–**I**) at 4, 8, 12, 24, and 48 h post-infection. All immunization trials potentiated the clearance of bacterial burdens in the tissues of immunized mice compared to the control non-immunized mice. Tissues were collected from three mice at each time interval in each group. Immunization with the recombinant proteins pool led to a significant reduction in bioburdens of liver tissues at all time intervals (**A**). Spleen bacterial burdens were significantly reduced at 12 h post-challenge (**B**), and kidneys bacterial burdens were significantly reduced after 8, 12, 24, and 48 h (**C**). Immunization with the capsular polysaccharides pool led to a significant reduction in bioburdens of liver tissues at time intervals 4, 8, 12, and 48 h (**D**), at 12 and 24 h in spleen tissues (**E**), and at 8 and 12 h in case of kidneys (**F**). Immunization with the pentavalent pool potentiated a significant reduction in bioburdens of liver tissues at all time intervals **(G)**, in the spleen at 12 h post-challenge (**H**), while in kidneys bacterial burdens were significantly reduced after 8, 12, 24 and 48 h of bacterial challenge (**I**). No bacterial burdens were detected in the tissues of negative control mice. * *P* < 0.05, ** *P* < 0.01 and *** *P* < 0.001

All immunization trials helped the challenged mice to eliminate bacterial loads in their tissues at all time intervals compared to the control non-immunized group, while no bacterial burdens were detected in the tissues of negative control mice. For immunization using the pool of recombinant proteins (Wza and YiaD), a significant reduction in bioburdens was detected in liver tissues at all time intervals (Fig. 5A), while bacterial burdens in the spleen were significantly lower at 12 h post-challenge of immunized mice (Fig. 5B). In kidneys, bacterial burdens were significantly lower after 8, 12, 24 and 48 h following the bacterial challenge (Fig. 5C).

For the capsular polysaccharides pool, a significant reduction in bioburdens was detected in liver tissues at time intervals of 4, 8, 12, and 48 h post-challenge compared to the control non-immunized mice (Fig. 5D). While in the spleen bacterial burdens were significantly reduced at 12 and 24 h post-challenge (Fig. 5E). In kidneys, bacterial burdens were significantly lower after 8 and 12 h of bacterial challenge compared to non-immunized mice (Fig. 5F).

Immunization with the pentavalent pool assisted mice to significantly reduce the bacterial loads in their liver tissues at all time intervals post their challenge (Fig. 5G). However, bioburdens were significantly lower only after 12 h following the bacterial challenge in spleens of immunized mice compared to the control non-immunized group (Fig. 5H). In kidneys, bacterial burdens were significantly reduced after 8, 12, 24, and 48 h of bacterial challenge (Fig. 5I).

### Histopathological examination of mice tissues

**Fig. 6:**
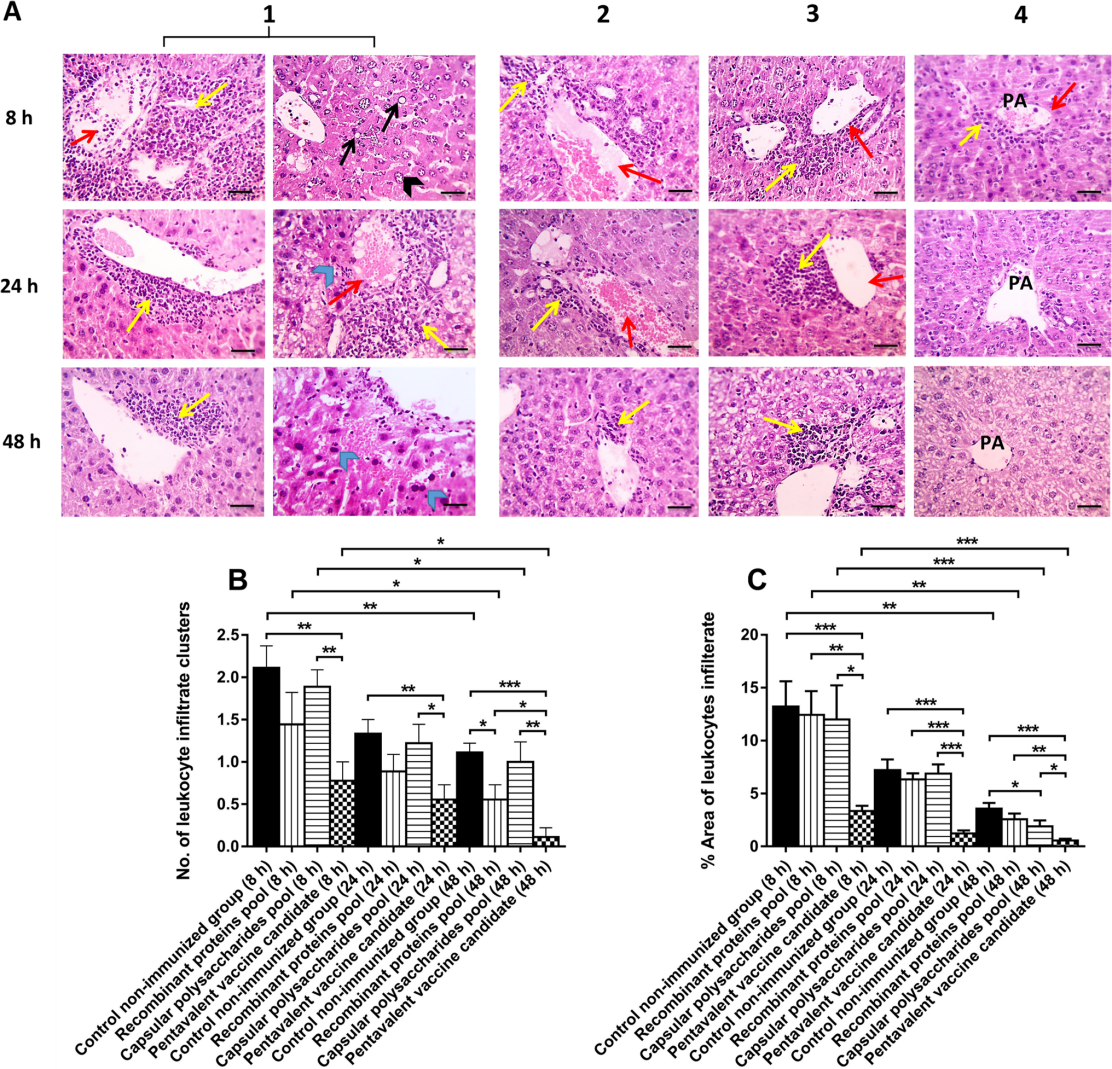
Histopathology of liver tissues following immunization and challenging experiments in mice. **(A)** represents microscopic pictures of H&E stained liver sections of the control non-immunized mice (1) and immunized mice with either recombinant proteins pool (2), capsular polysaccharides pool (3), or pentavalent pool (4). Liver tissues were collected from three mice at each time interval in each group. In control, non-immunized mice liver sections showed severe congestion (red arrows) with perivascular inflammation (yellow arrows). Nuclear vacuolization of hepatocytes (black arrows) with the presence of basophilic bacteria in hepatic sinusoids (black arrowheads) appeared after 8 h only. Congestion and perivascular inflammation slightly decreased at 24 and 48 h post-challenge accompanied with nuclear pyknosis (blue arrowheads). Liver sections of immunized mice with either recombinant proteins or polysaccharides pool showed congestion (red arrows) and perivascular inflammation (yellow arrow) at 8 and 24 h post-challenge, the degree of inflammation decreased at 48 h time interval. Liver sections of immunized mice with pentavalent pool showed mild perivascular inflammation (yellow arrow) at 8 and 24 h time intervals that subsides at 48 h post-challenge. Liver sections of control uninfected mice showed normal hepatocytes arranged in radiating plates around the central vein with normal portal areas and sinusoids. In **B** and **C**, immunization with the pentavalent pool significantly reduced inflammation of liver tissues represented by a significant decrease in both the number of leukocyte infiltrate clusters (**B**) and the percent area of leukocyte infiltrate (**C**) compared to all other mice groups. The results were calculated as means (± standard error of the mean) of triplicates. * *P* < 0.05, ** *P* < 0.01 and *** *P* < 0.001. High magnification × : 400 bar 50

Negative control uninfected mice exhibited normal hepatocytes arranged in radiating plates around central vein (CV) with normal portal areas (PA) and sinusoids. No infiltration of leukocytes into different tissue sections was observed. Liver tissues of control non-immunized challenged mice showed severe congestion with perivascular inflammation. Moreover, nuclear vacuolization of hepatocytes with the presence of basophilic bacteria in hepatic sinusoids appeared at 8 h only, following infection. Congestion and perivascular inflammation slightly decreased at 24 and 48 h, post-challenge of mice, accompanied with nuclear pyknosis (Fig. 6A (1)).

Following the challenge of mice immunized with the recombinant proteins pool, liver tissues also exhibited congestion and perivascular inflammation at 8 and 24 h time intervals. The inflammation degree was then decreased at 48 h post-challenge (Fig. 6A (2)). Additionally, the number of leukocyte infiltrate clusters was significantly lower in the case of immunized mice after 48 h post-challenge when compared to the control non-immunized mice (Fig. 6B). Moreover, the percent area of leukocyte infiltrate appeared lower than that in control non-immunized mice at each time interval (Fig. 6C).

In the case of capsular polysaccharides pool immunization, congestion and perivascular inflammation were observed in liver sections after 8 and 24 h of the bacterial challenge; however, the degree of inflammation decreased at 48 h post-infection (Fig. 6A (3)). Additionally, the number of leukocyte infiltrate clusters appeared lower than that in control non-immunized mice at each time interval (Fig. 6B). Moreover, the percent area of leukocyte infiltrate in immunized mice appeared lower than that of the control non-immunized mice at each time interval, however, it was significantly significant at 48 h post-infection (Fig. 6C).

Liver tissues of immunized mice with the pentavalent pool showed mild perivascular inflammation at 8 and 24 h time intervals that subsided at 48 h post-challenge (Fig. 6A (4)). Moreover, leukocyte infiltration rate, represented by both the number of leukocyte infiltrate clusters and the percent area of leukocyte infiltrate, appeared significantly lower than those recorded in any other group, including the control non-immunized mouse group (Fig. 6B and C).

All mice groups (immunized and control non-immunized) showed a significant reduction in leukocyte infiltration rates, represented by both the number of leukocyte infiltrate clusters and the percent area of leukocyte infiltrate, at 48 h post-challenge compared to their values at 8 h time interval (Fig. 6B and C).

## Discussion

*A. baumannii* is considered one of the most virulent and infectious organisms that have an increased ability to both evade host immune response and resist various classes of antibiotics, leading to life-threatening pneumonia and bacteremia (Wong and Nielsen 2017). Moreover, improper use of antibiotics has led to the emergence of MDR and XDR strains where even carbapenems, colistin, tigecycline, and combination antibiotic therapy may not be effective, thus alternative strategies are urgently required (Nowak and Paluchowska 2016). Vaccine development has recently received much attention to protect against *A. baumannii*. However, as far as we know, there is still no licensed vaccine approved against *A. baumannii*, where the vast diversity among its strains remains to be the major challenge in the development of an effective vaccine (Gellings and Wilkins 2020; Yang and Lou 2017). Herein, we report the development of a novel pentavalent vaccine candidate composed of two new recombinant proteins (Wza and YiaD) and a pool of capsular polysaccharides isolated from three *A. baumannii* clinical isolates. Immunization with this vaccine candidate completely protected mice with 100% survival against all tested clinical bacterial isolates.

One of the major virulence determinants of *A. baumannii* is the presence of OMPs representing feasible targets for immunization. Presence of the dense polysaccharide capsule that might overshadow outer membrane antigens from immune recognition has limited their use as vaccine candidates (Wang-Lin et al. 2017). Nevertheless, identification of the protruding protein portions extending through the capsule to the external environment could overcome that limitation (Gellings and Wilkins 2020). To be an effective vaccine candidate, it should be surface exposed, conserved among different strains of *A. baumannii*, highly prevalent, immunogenic, and able to illicit a reproducible protective immune response in population (Ahmad and Tawfik 2016). Many *in silico* prediction tools are currently being used to predict protein epitopes fulfilling vaccine candidate requirements, such as, reverse vaccinology alone or combined with comparative genomic analysis, *in vitro* proteomic analysis, and immunoproteomics (Chiang et al. 2015; Mujawar et al. 2019).

OmpA family is one of the most conserved OMPs in *A. baumannii* that has been implicated in bacterial virulence, adhesion, invasion, and interaction with surface receptors on host cells. Members of this family represent very promising vaccine candidates (Ansari et al. 2019). Immunization with OmpA was previously reported to significantly increase the survival rates of mice and decrease bioburdens in their tissues post-challenge with *A. baumannii* (Luo et al. 2012; Zhang et al. 2016). Interestingly, OmpA family proteins have been formerly reported to be able to induce protective immunity against variable bacteria including *Pseudomonas aeruginosa*, *Shigella flexneri*, *Chlamydia abortus*, *Escherichia coli*, and *Klebsiella pneumonia* (Ayalew et al. 2011; Hounsome et al. 2011; Jeannin et al. 2002; Lei et al. 2019; Pore and Chakrabarti 2013; Tang et al. 2017). However, OmpA provided insufficient protection levels against *A. baumannii* increasing the urge to identify additional novel protective antigens (Chen 2015).

The outer membrane protein Wza (formerly known as EpsA) is an OMP that belongs to the capsule biosynthesis gene cluster and is implicated in lipopolysaccharides export (Ahmad and Azam 2018), while YiaD (formerly known as ABAYE2931) is an OMP belonging to OmpA family (Chiang et al. 2015). Both proteins (Wza and YiaD) are antigenic, non-allergen, highly conserved, and strongly activating of both humoral and cell-mediated immunity (Ahmad and Azam 2018; Chiang and Sung 2015). In the present study, we investigated for the first time, as far as we know, the ability of both Wza and YiaD to induce protective immune responses against *A. baumannii*. From the standard ATCC 19606 strain, we expressed the full-length coding sequence of YiaD; however, in the case of Wza, we only expressed a segment of the coding sequence that contained the antigenic epitope “LQNNTRRMK”. It was previously reported that immunization with the antigenic epitopes only of some proteins, and not the whole protein sequence, may be sufficient to give protection against *A. baumannii* (Du et al. 2021). Both Wza and YiaD successfully induced significantly high antibody titers following subcutaneous immunization of mice. An exceptional cross-reactivity over 97% with more than 90% of binding was detected between individual immune sera of Wza and YiaD and 100 different clinical isolates using ELISA. Moreover, our *in vivo* studies proved that active immunization of mice with either recombinant Wza or YiaD alone, significantly protected mice against the invading standard strain with 70% or 60% survival rates, respectively, compared to only 20% in the case of the non-immunized control group. Similarly, it was reported by Singh R and coworkers (2018), that immunizing mice with FilF, an OMP, was associated with 50% protection against the standard ATCC 19606 strain (Singh et al. 2016).

We extended our survival experiments to include 3 representative clinical isolates based on their binding levels with immune sera, where clinical isolates No. 62 and No. 100 showed higher binding than that obtained with the standard strain from which the protein immunogens were derived (more than 100% binding). Binding of clinical isolates No. 62 and No. 100 was similar to binding of 95% and 86% of isolates for Wza and YiaD immune sera, respectively. On the other hand, clinical isolate No. 63 showed less binding degree of 89% and 90%with immune sera against Wza and YiaD, respectively. This binding was similar to 5% and 14% of isolates for Wza and YiaD immune sera, respectively. Immunization with Wza or YiaD was associated with survival rates ranging from 60 to 70% for clinical isolates No. 62 and No. 100. However, neither Wza nor YiaD immunized mice were protected against their challenge with the clinical isolate No. 63. Similar or even higher protection rates (ranging from 50 to 100% survival) were previously reported by other researchers following immunization of mice with other OMPs including OmpA, Omp22, and OmpW against few selected clinical isolates (Huang et al. 2015, 2016; Luo and Lin 2012).

The use of dual-component vaccination strategy has been formerly evaluated and shown to be successful (Bolourchi et al. 2019; Ramezanalizadeh et al. 2020). In one study, OmpA was combined with the secreted serine protease PKF in an antigen cocktail. This cocktail protected mice that showed increased clearance levels of *A. baumannii* bioburdens and increased survival rate to 85% compared to 80% and 75% survival rates in the case of either PKF or OmpA, respectively (Bolourchi and Shahcheraghi 2019). In another study, combined FimA and CsuA/B acted synergistically on immunized mice leading to increased survival up to 60% compared to FimA (50%) or CsuA/B (35%), when given alone (Ramezanalizadeh and Owlia 2020). Those studies suggested that a cocktail of conserved surface proteins could represent a highly effective vaccine against the majority of *A. baumannii* clinical isolates. This is not particularly surprising given that there are many FDA-approved multicomponent vaccines on the market today, including the Diphtheria, Tetanus, and Pertussis (DTaP) and Meningitis B (MenB) vaccines (Gellings and Wilkins 2020). In our study, immunization with the recombinant proteins cocktail (Wza and YiaD pool) led to a synergistic immune response that protected immunized mice with survival rates ranging from 90% to even 100% against all tested *A. baumannii* standard and clinical isolates. Additionally, more than 80% antibody binding was observed between the immune serum of immunized mice with the recombinant proteins pool and all tested clinical isolates. Such a vaccination strategy succeeded to extend the spectrum of protection to include clinical isolate No. 63, where the survival rate in immunized mice reached 90%. Moreover, bioburden levels and the number of leukocyte infiltrate clusters were significantly reduced in immunized mice tissues post-challenge with *A. baumannii*.

Outer polysaccharide capsules also represented plausible targets for immunization against *A. baumannii* (Gellings and Wilkins 2020). Passive immunization using monoclonal antibodies against *A. baumannii* K1 capsular polysaccharide was formerly reported to decrease bacterial burdens in a soft-tissue infection model (Russo and Beanan 2013). Additionally, antibodies against capsular polysaccharides from the drug‐resistant clinical strain SK44 reduced post‐infection bioburdens and provided 55% protection against *A. baumannii* in a murine pneumonia model (Yang and Lou 2017). However, the vast diversity in capsule composition, with over 100 distinct capsule types identified to date, among *A. baumannii* populations has hindered its use as a vaccine candidate unless clinicians were able to rapidly identify and target the correct capsule profile (Wyres et al. 2020). Herein, the capsular polysaccharides of the standard ATCC 19606 strain completely protected immunized mice with 100% survival rate against the same standard strain. Even though, the enormous varieties in the composition of capsular polysaccharides among different clinical *A. baumannii* isolates have limited their cross-reactivity with the immune serum developed against the capsule of the standard strain. To overcome this issue, we tried a pool of capsular polysaccharides prepared from three clinical isolates (No. 51, No. 63, and No. 76) that showed variable similarity degrees in capsule composition with all tested clinical isolates. These similarities were estimated through different levels of cross-reactivity between tested clinical isolates and antisera formed against either each capsule alone or their pool together. Individual capsular polysaccharides from five different clinical isolates were initially tried in immunization, where isolates No. 79 and No. 100 were excluded as no cross-reactivity was observed between their immune sera and the tested clinical isolates. However, variable degrees of cross-reactivity were observed between tested clinical isolates and immune sera of mice that received capsular polysaccharides of either isolate No. 51 or No. 63. Despite the low levels of cross-reactivity, capsular polysaccharides of clinical isolate No. 76 were added in our polysaccharides pool due its cross-reactivity with all uncovered clinical isolates by capsular polysaccharides of either isolate No. 51 or No. 63.

Furthermore, we have evaluated the protective efficacy of the capsular polysaccharides pool in mice. Immunization with this pool has protected the challenged mice with a 70% survival rate against the standard strain and with survival rates ranging from 80 to 90% against all tested clinical isolates (No. 62, No. 63, and No. 100). Moreover, immunization of mice with the capsular polysaccharides pool managed not only to significantly assist mice to clear their tissues from the bacterial loads, but also to significantly decrease inflammation levels, through decreased percent area of the leukocyte infiltrate after 48 h, in their liver tissues compared to the control non-immunized mice. These results were consistent with Russo and coworkers (2013), who reported that immunization using K1 capsular polysaccharide decreased K1-positive strain bioburdens in mice while having no effect on K1-negative strains (Russo and Beanan 2013).

A major drawback of using capsular polysaccharides as vaccine candidates remains to be the T cell-independent immune response, where only B cells are activated (Feldman et al. 2019). Interestingly, combining polysaccharides with recombinant proteins could generate a T cell-dependent antigen, where both B and T cells are involved in the immune response required to generate a successful immune memory (Li et al. 2021). To improve the overall efficacies of the different tested vaccine candidates, we decided to formulate and evaluate a new pentavalent vaccine composed of both recombinant proteins and capsular polysaccharides pools. Surprisingly, the pentavalent pool succeeded to completely protect immunized mice with 100% survival rates against all the tested *A. baumannii* whether the standard strain or the clinical isolates (No. 62, No. 63, and No. 100). Additionally, the pentavalent pool managed not only to significantly increase the clearance of bacterial loads in immunized mice tissues, but also to significantly reduce inflammation of their liver tissues. It was also observed that the pentavalent pool was superior to other tested pools (either recombinant proteins or capsular polysaccharides pools) regarding decreased inflammation of liver tissues in immunized mice. Both the number of leukocyte infiltrate clusters and the percent area of leukocyte infiltrate were significantly reduced in liver tissues of immunized mice with the pentavalent pool compared to all other mice groups. Interestingly, Li and coworkers (2021) reported the efficacy of such a cocktail of both capsular polysaccharides and recombinant proteins. They showed that a conjugate vaccine formed of capsular polysaccharides of *A. baumannii,* and the recombinant cholera toxin B subunit (CTB4573C) could protect immunized mice with survival rates ranging from 70 to 100% compared to only 20% and 30% in case of immunization using either CTB or capsular polysaccharides alone. The glycoconjugate vaccine also succeeded to decrease bacterial tissue loads in immunized mice compared to the non-immunized groups (Li and Pan 2021). However, the capsular polysaccharides-CTB conjugate vaccine still needs a further assessment as an immunogen against other clinical *A. baumannii* isolates, as it was only tested against one standard strain and one clinical *A. baumannii* isolate.

In our future work, we would recommend the evaluation of our pentavalent pool against other isolates of *A. baumannii* from different geographic sources worldwide. Throughout our study, we have observed that isolated capsular polysaccharides from the standard ATCC 19606 strain, firstly isolated in the USA (Tsubouchi et al. 2020), were highly variable in composition than those isolated from the clinical isolates that were collected from Egypt. This variability was confirmed by low levels of cross-reactivity between the immune serum of mice that received capsular polysaccharides of the standard strain alone, and all tested clinical isolates, suggesting that *A. baumannii* capsules could respond differently according to their geographical source and surrounding environmental conditions. However, immunized mice with the polysaccharides pool were protected following the challenge with the standard strain despite the absence of the standard strain polysaccharides from the immunizing polysaccharides pool.

In summary, for the first time, we introduced several new promising vaccine candidates against *A. baumannii* infections, including two recombinant proteins Wza and YiaD, a pool of both Wza and YiaD, a pool of three capsular polysaccharides, and a pentavalent pool of Wza, YiaD, and three capsular polysaccharides. Combining both recombinant proteins and capsular polysaccharides in our currently investigated pentavalent pool has managed to overcome the disadvantages of using either of them alone, and has enhanced the overall vaccine efficacy. This novel pentavalent vaccine candidate managed to completely protect immunized mice against challenges with all tested *A. baumannii* including the standard ATCC 19606 strain and the clinical isolates. This was coupled with a significant reduction in bioburden and inflammation levels in tissues of immunized mice compared to non-immunized control mice.

## Supplementary Information

Below is the link to the electronic supplementary material.

Supplementary file1 (PDF 1130 KB)

## Data Availability

All data generated and analyzed during this study are included in this article and its supplementary information files.
