# Supplementary material for: A novel pentavalent vaccine candidate completely protects against Acinetobacter baumannii in a mouse model of peritonitis
Source: Appl Microbiol Biotechnol. 2022 Nov 19;106(24):8151–67. doi: 10.1007/s00253-022-12231-3 (PMC9676856; doi:10.1007/s00253-022-12231-3)
Supplement: Supplementary file 1 — Supplementary file1 (PDF 1130 KB) [file 253_2022_12231_MOESM1_ESM.pdf]

Applied Microbiology and Biotechnology

A novel pentavalent vaccine candidate completely protects against *Acinetobacter baumannii* in a mouse model of peritonitis

Yomna A. Hagag, Heba Shehta Said, Hany I. Kenawy, and Ramadan Hassan

Department of Microbiology and Immunology, Faculty of Pharmacy,
Mansoura University, Mansoura 35516, Egypt.

Corresponding authors:

Heba Shehta Said, Ph.D.

Address: Department of Microbiology and Immunology, Faculty of Pharmacy, Mansoura University, Mansoura 35516, Egypt.

E-mail: hebashehta@mans.edu.eg

ORCID ID: https://orcid.org/0000-0002-4456-6001

Hany I. Kenawy, Ph.D.

Address: Department of Microbiology and Immunology, Faculty of Pharmacy, Mansoura University, Mansoura 35516, Egypt.

E-mail: hanykenawy@mans.edu.eg

ORCID ID: https://orcid.org/0000-0002-8548-6649

Supplementary Figures:

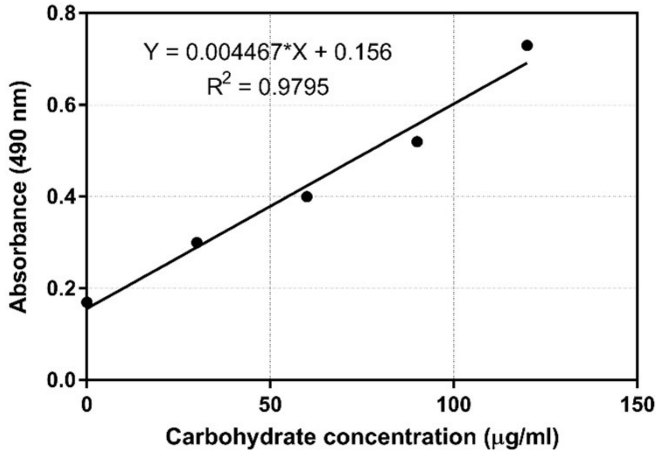

Fig. S1. Standard calibration curve of absorbance (at 405 nm) versus carbohydrate concentration. Serial dilutions of carbohydrate stock (1:1 mixture of 0.5 mg/ml each of sucrose and fructose) were assayed for concentration where, 200 µl of each dilution were swirled with 200 µl of 5% phenol and 1 ml of 93% sulfuric acid. Color was allowed to develop for 10 min at room temperature with extra gentle swirling every 2-3 minutes. Optical density (OD_{490}) was then measured, and standard calibration curve was obtained with R^2=0.9795.

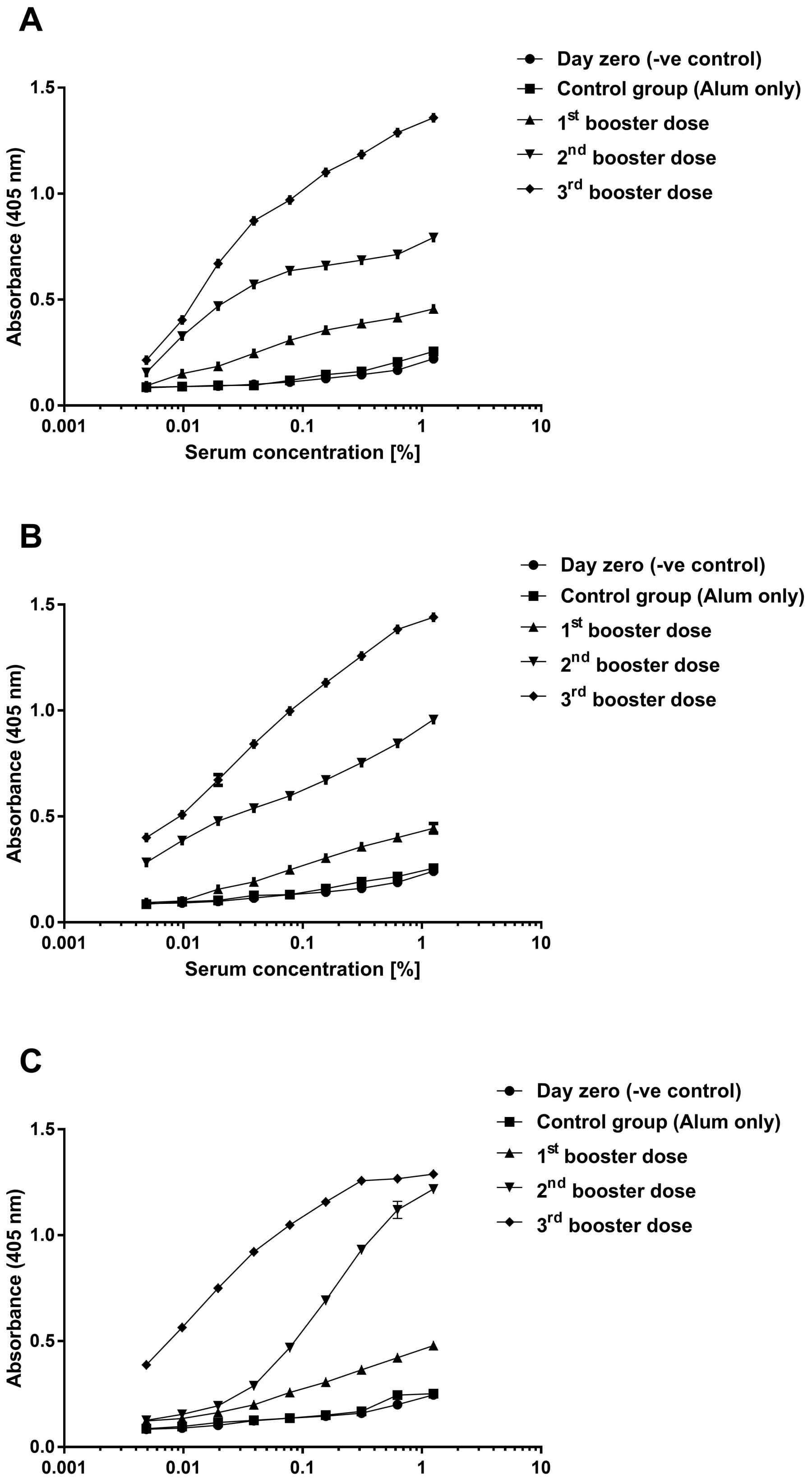

A
Day zero (-ve control)
Control group (Alum only)
1st booster dose
2nd booster dose
3rd booster dose
Absorbance (405 nm)
1.5
1.0
0.5
0.0
0.001
0.01
0.1
1
10
Serum concentration [%]
B
Day zero (-ve control)
Control group (Alum only)
1st booster dose
2nd booster dose
3rd booster dose
Absorbance (405 nm)
Serum concentration [%]
C
Day zero (-ve control)
Control group (Alum only)
1st booster dose
2nd booster dose
3rd booster dose
Absorbance (405 nm)
Serum concentration [%]

Fig. S2. Determination of antibody titre against recombinant proteins, either wza (A) or yiaD (B), and against capsular polysaccharides of standard ATCC 19606 strain (C) in immunized mice sera. Three female BALB/c mice were immunized with either wza or yiaD recombinant proteins or capsular polysaccharides of the standard *A. baumannii* strain ATCC 19606 adjuvanted with alum. Mice received four vaccination doses at days 0, 7, 14, and 21. Immune sera were collected at day zero (as a negative control), 14, 21, and 28 from immunized mice, in addition to the control group that received alum only. ELISA results revealed significant increase in antibody titre in immune sera isolated from immunized mice upon injecting booster immunization doses. Control group that received alum only showed no immune response against immunogens.

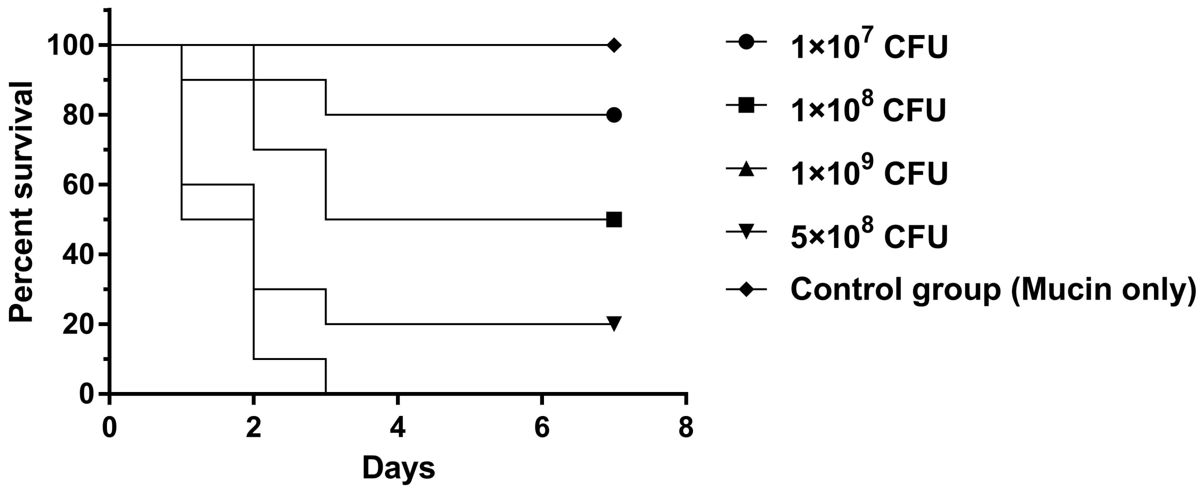

Fig. S3. Determination of the challenging bacterial dose (LD_{80}) of *A. baumannii.* Female BALB/c mice groups (n=10/ group) were challenged intra-peritoneally with different doses of bacteria. Infectious doses of 1×10^7 and 1×10^8 CFU/mouse of the standard ATCC 19606 strain led to survival of more than 40% of the challenged mice. Increasing the infectious dose to 5×10^8 CFU/mouse resulted in a reduction of survival to 20%. A dose of 1×10^9 CFU/mouse was associated with no survivors among all the challenged mice. Survival rate was 100% among control mice that received mucin only.
